# Effect of Intensive Face Yoga on Facial Muscles Tonus, Stiffness, and Elasticity in Middle-Aged Women: A Pre-Experimental Clinical Trial

**DOI:** 10.3390/medicina61050840

**Published:** 2025-05-02

**Authors:** Hazel Çelik Güzel, Şule Keçelioğlu, Ahmet Kurtoğlu, Safaa M. Elkholi

**Affiliations:** 1 Department of Therapy and Rehabilitation, Health Services Vocational School, Bandırma Onyedi Eylül University, Balıkesir 10200, Türkiye; 2 Department of Physiotherapy and Rehabilitation, Faculty of Health Sciences, Bandırma Onyedi Eylül University, Balıkesir 10200, Türkiye; 3 Department of Coaching Education, Faculty of Sport Science, Bandırma Onyedi Eylül University, Balıkesir 10200, Türkiye; 4 Department of Rehabilitation Sciences, College of Health and Rehabilitation Sciences, Princess Nourah bint Abdulrahman University, P.O. Box 84428, Riyadh 11671, Saudi Arabia

**Keywords:** anti-aging, elasticity, muscle tonus, stiffness, yoga

## Abstract

*Background and Objectives*: The effects of face yoga, which continues to be popular as an anti-aging technique, on facial muscles in relation to aging represent an area of interest. The aim of this study was to investigate the effect of 8 weeks of intensive face yoga on facial muscles’ tonus, stiffness, and elasticity in middle-aged women. *Materials and Methods*: Twelve female participants with a mean age of 49.75 ± 3.76 were included in this pre-experimental clinical trial. Face yoga was applied to the participants for 8 weeks, 2 days a week face-to-face, and 5 days a week as a home program. At the beginning and the end of 8 weeks, the tonus, stiffness, and elasticity of the participants’ facial muscles were evaluated with the Myoton^®^PRO device. *Results*: Following the face yoga program, the tonus and stiffness of the frontalis (*p* = 0.008, *p* = 0.002), corrugator supercilii (*p* = 0.008, *p* = 0.019), orbicularis oculi (*p* = 0.023, *p* = 0.034), and orbicularis oris (*p* = 0.007, *p* = 0.015) muscles decreased significantly, while the tonus and stiffness of the buccinator (*p* = 0.008, *p* = 0.002) and digastric (*p* = 0.008, *p* = 0.023) muscles increased. Elasticity values increased in all evaluated facial muscles (*p* = 0.045, *p* = 0.045, *p* = 0.034, *p* = 0.023, *p* = 0.028, *p* = 0.005, respectively). *Conclusions*: The results reveal that face yoga has different effects depending on the physiological structure and function of the muscles and positively affects connective tissue. Future studies should reproduce the results presented here to further our understanding of the effects of face yoga.

## 1. Introduction

Aging is an inevitable phase of human life that leaves scars on the face [1]. It causes changes in many different layers of the face, such as the skin, bone, muscle, and adipose tissue. The changes in bone structure brought about by the aging process cause soft tissues to sag; atrophy in adipose tissues causes the cheeks to collapse and facial contours to flatten; the loss of strength in facial muscles, repetitive muscle contractions, and changes in muscle tonus cause wrinkles to form and facial expression to change; the thinning and decreased elasticity of the skin causes wrinkles to form [2]. Some mechanisms explain the changes seen in facial muscles with aging. Accordingly, it is stated anatomically that deep adipose tissue in the face is located in large and localized clusters under the muscles. In contrast, superficial adipose tissue shows a thin, regular arrangement between the skin and muscle. While the muscle at rest in young individuals has a convex appearance with deep adipose tissue, over time, the muscle becomes flatter and shorter, and its resting tonus increases and loses its convexity, considering that the contraction of the muscles is repeated millions of times during facial expressions. This leads to a decrease in deep adipose tissue and an increase in superficial adipose tissue. In other words, deep adipose tissue is pushed up and down by muscle contraction, and superficial adipose tissue increases [3]. It is also noted that although the facial muscles lose strength, they cause exaggerated expressions due to their relative traction angles in less resistant tissues. Therefore, repetitive muscle contractions create superficial and deep dynamic wrinkles [2]. For example, the orbicularis oris muscle causes tense, puckered lips as a result of the thickening of connective tissue with aging; the repetitive contraction of the lateral orbicularis oculi muscles causes crow’s feet lines; the contraction of the corrugator supercilii and procerus muscles causes transverse lines of the nasal root; the overactivity of the depressor anguli oris and platysma muscles causes labiomandibular folds; and the repetitive movement of the mentalis muscle causes the labiomental fold to become prominent. The decreased activation of the frontalis muscle and normal or the increased tonus of the orbicularis oculi muscles may also be associated with eyebrow drooping [2].

Several practical approaches to treating facial signs of aging include botulinum toxin injections, dermal fillers, and surgical methods [4]. However, these methods have limitations due to their invasiveness, high costs, and potential side effects. Although botulinum toxin injections are widely used to treat wrinkles, they can cause local side effects such as erythema, edema, and transient eyelid drooping [5]. More importantly, long-term and repeated administration increases the risk of muscle atrophy, which can result in muscle weakening and volume loss [6,7]. At this point, it is stated that face yoga, which functions as a noninvasive alternative to Botox and surgery and has emerged as anti-aging, can contribute to increased skin elasticity, the stimulation of facial muscles, the softening of facial lines, lifting the skin, increased blood circulation, the renewal of skin cells, increased collagen production, and reduced tension in the face [8,9]. On the other hand, it is hypothesized that face yoga can stimulate muscle growth and improve the appearance of facial skin in response to the loss of facial fat and muscle mass with aging [10]. Face yoga is based on facial exercises and breathing techniques and includes a series of practices such as stretching exercises, relaxation techniques, posture exercises with core stabilization, and classical massage therapy [11]. It contributes to the stimulation of different facial muscles such as the orbicularis oculi, orbicularis oris, mentalis, frontalis, zygomaticus major, platysma [9]. For example, an exercise to reduce vertical wrinkles above the upper lip trains the orbicularis oris muscle; an exercise to reduce nasolabial folds trains the orbicularis oris and zygomaticus minor muscles; an exercise to reduce a double-chin and achieve a more obvious jawline trains the masseter, sternocleidomastoid, and mylohyoid muscles; and an exercise to reduce wrinkles on the forehead trains the frontalis muscle [1]. However, the literature states that studies on the effectiveness of facial exercises are insufficient [12], and it is noteworthy that this field is just beginning to sprout. In this context, our study aimed to investigate the effect of 8 weeks of intensive face yoga on the facial muscles’ tonus, stiffness, and elasticity in middle-aged women.

## 2. Materials and Methods

### 2.1. Study Design

This study was designed as a pre-experimental clinical trial and conducted between June 2024 and January 2025, according to the Declaration of Helsinki Recommendations, with ethical approval from the Bandırma Onyedi Eylül University Health Sciences Non-Interventional Research Ethics Committee (Date = 17 May 2024, Approval Number: 2024-113). The study’s Clinical Trials Number was NCT06765408.

### 2.2. Study Participants

The population of the study comprised staff from an academic institution. The study sample, using convenience sampling, consisted of female staff between the ages of 45 and 55 (middle-aged women), from whom voluntary consent was obtained. Subjects who had undergone Botox application to the facial area in the last 6 months, the regular use of analgesic and anti-inflammatory drugs, the presence of infection or tumoral structures in the facial structures, temporomandibular joint (TMJ) disk displacement and joint degeneration, cervical or TMJ fracture, systemic disease, along with individuals with musculoskeletal problems with evidence of a specific pathological condition, who had undergone any surgical operation related to cervical or TMJ problems, who had received cervical or TMJ-related physiotherapy and rehabilitation services in the last 6 months, who had facial paralysis, and who had a diagnosed psychiatric illness were excluded from the study. The call for participation in the study was provided using the poster method. After verbal and written information was given to the participants who voluntarily participated in the study, an informed consent form was obtained from the participants.

For the post hoc power analysis in the study, buccinator muscle tone was selected as the primary outcome measure since (1) the sensorimotor connections between the trigeminal and facial nerves are evident in the buccinator muscle and potentially play a role in facial muscle proprioception [13], (2) there is a significant loss of elasticity and stiffness of the buccinator muscle with age (max 41–50 years) [14], (3) it is the most active muscle on the face during facial expressions [15]. For these reasons, the buccinator is predestined to be a muscle with a high potential to respond to face yoga. The study’s power was calculated as 85% with a 95% confidence interval, considering the buccinator muscle’s effect size (r = 0.86).

### 2.3. Outcome Measures

In the study, a Descriptive Data Form was used for the demographic characteristics of the participants, and the Myoton^®^PRO device (Myoton Ltd., Myoton AS, Tallinn, Estonia) was used for the facial muscles’ tonus, stiffness, and elasticity. The primary outcome of the study was the tonus of the facial muscles, and the secondary outcomes were the stiffness and elasticity of the facial muscles. The frontalis, corrugator supercilii, orbicularis oculi, orbicularis oris, buccinator, and digastric muscles were evaluated.

### 2.4. Descriptive Data Form

This form, created by researchers, includes questions regarding age (years), height (centimeter, cm), weight (kilogram, kg), Body Mass Index (BMI, kg/m^2^), current medications, Botox application to the face within the last 6 months, the presence of an infection or tumor in the structures of the face, the presence of TMJ disk displacement and joint degeneration, cervical or TMJ fracture, systemic disease, a musculoskeletal system problem with a proven specific pathological condition, any surgical operation related to a cervical or TMJ problem, receiving cervical or TMJ-related physiotherapy and rehabilitation services within less than 6 months, having facial paralysis, and having a diagnosed psychiatric disease.

#### Myoton^®^PRO Device

**Figure 1:**
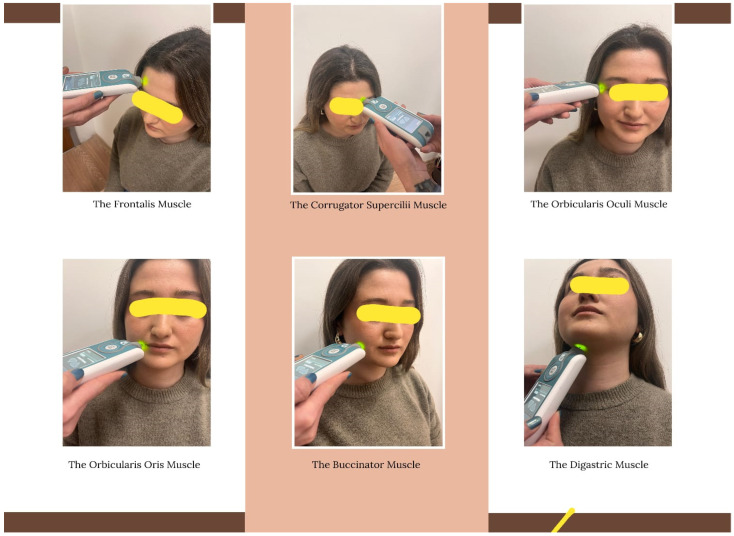
Evaluation of the frontalis, corrugator supercilii, orbicularis oculi, orbicularis oris, buccinator, and digastric facial muscles with the Myoton^®^PRO device.

Myoton^®^PRO (Myoton Ltd., Myoton AS, Estonia), a portable digital palpation device, was used to objectively assess the biomechanical properties of the participants’ facial muscles, such as tonus, stiffness, and elasticity. When the tip of the probe (3 mm diameter, polycarbonate) is applied perpendicular to the surface above the muscle under test at a constant preload (0.18 N) to pre-compress the subcutaneous tissues, it quickly delivers a short mechanical impulse (15 ms), triggering a short electromagnetic impulse of constant strength (0.4 N) that is transmitted to the tissue. The mechanical impulse from the device’s probe causes elastic deformation in the measured muscle tissues. It produces naturally damped oscillations recorded by the frictionless and sensitive accelerometer sensors at the other end of the probe as the tissue returns to its original shape. This allows the simultaneous calculation of tissue parameters, including the mechanical properties of the muscle such as the tonus (F-frequency; Hz), stiffness (S-N/m), and elasticity (D-LD) [16,17]. The participants’ frontalis, corrugator supercilii, orbicularis oculi, orbicularis oris, buccinator, and digastric facial muscles were evaluated bilaterally, right and left, in the sitting position. For each muscle, three measurements were taken from the right and left sides at one-second intervals, and the mean value of each measurement was recorded. In the analysis, the mean values of each muscle from the right and left sides were averaged. The midpoint of the muscle belly/motor point was taken as the measurement region [18], and the muscle belly was determined according to its anatomical location [19,20,21]. The evaluation of the frontalis, corrugator supercilii, orbicularis oculi, orbicularis oris, buccinator, and digastric facial muscles with the Myoton^®^PRO device is shown in Figure 1.

### 2.5. Procedure

After the participants who volunteered to participate in the study signed the informed consent form, they were asked to complete the Descriptive Data Form. The tonus, stiffness, and elasticity of the frontalis, corrugator supercili, orbicularis oculii, orbicularis oris, buccinator, and digastric facial muscles of the participants who met the study inclusion criteria according to the Descriptive Data Form were evaluated with the Myoton^®^PRO device. Then, the participants were included in a face yoga program with a physiotherapist who had received face yoga training and was an expert in the field; for 8 weeks, they had face-to-face sessions 2 days a week and a home program 5 days a week, with each session lasting an average of 30 min. Therefore, the face yoga program was implemented as 16 face-to-face sessions and 40 home programs. A WhatsApp group was created to monitor the participants’ compliance with the home program, and participants were informed and reminded every day. In addition, participants were asked to fill out follow-up charts to ensure compliance with the home program. At the end of 8 weeks, the tonus, stiffness, and elasticity of the frontalis, corrugator supercilii, orbicularis oculi, orbicularis oris, buccinator, and digastric facial muscles were re-evaluated with the Myoton^®^PRO device.

The practiced face yoga program was selected from the exercises in the literature [22,23,24] by the researcher (HÇG), who received face yoga training, and the practiced face yoga program is given Table 1. In addition, the brochure prepared for the face yoga program is attached in the Supplementary Materials. A 10 s break was taken in normal breathing at each exercise transition. No scale (e.g., Borg) was used to assess participants’ subjective effort levels during the intervention. Instead, all participants practiced the standard face yoga protocol at the same intensity, and therapist observation monitored their compliance.

#### Statistical Analysis

SPSS for Windows version 23.0 software (IBM Corp., Armonk, NY, USA) was used for statistical analysis. In descriptive statistics, numerical variables with mean ± standard deviation (SD) and (minimum (min)–maximum (max)) values were given. The suitability of the data for normal distribution was determined according to skewness–kurtosis values. The Wilcoxon Signed-Rank Test was used to compare two dependent groups because the data were not normally distributed. Additionally, analysis results were given with effect sizes. The effect size indicates how much of the total variance in the dependent variable is explained by the independent variable or factor. The matched-pairs rank biserial correlation coefficient (r) is reported as an effect size due to its suitability for nonparametric data. This coefficient was calculated using the Wilcoxon test’s Z statistic and the sample size (N = matched pairs with zero differences) by the formula r = Z/√N. An effect size 0.20–0.50 was interpreted as “small”, 0.51–0.80 as “medium”, 0.81 and above as “large” [25].

## 3. Results

The mean age of the 12 female participants was 49.75 ± 3.76, ranging between 45 and 55 (middle-aged women). Their mean BMI was 28.32 ± 2.23 kg/m^2^, varying between 25.91 and 33.30 kg/m^2^.

**Figure 2:**
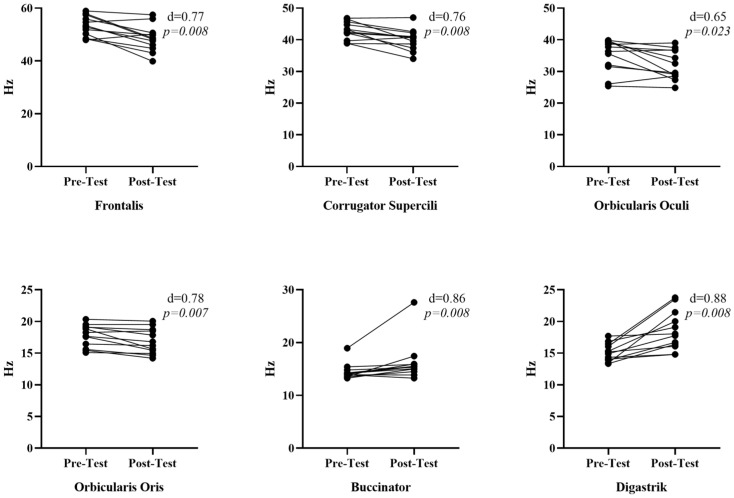
Comparison of facial muscle tonus values pre- and post-face yoga.

According to the data presented in Figure 2, when the tonus values (Hz) of facial muscles pre-and post-face yoga were compared, a significant decrease was observed in the tonus values of the frontalis [r = 0.77, *p* = 0.008, 95% CI [−10.4–2.2 Hz]], corrugator supercili [r = 0.76, *p* = 0.008, 95% CI [−7.25–1.05 Hz]], orbicularis oculi [r = 0.65, *p* = 0.023, 95% CI [−11.0–2.5 Hz]], and orbicularis oris [r = 0.78, *p* = 0.007, 95% CI [−3.2–0.25 Hz]] muscles. On the other hand, a significant increase was observed in the tonus values of the buccinator [r = 0.86, *p* = 0.008, 95% CI [−0.65–8.65 Hz]] and digastric [r = 0.88, *p* = 0.002, 95% CI [−0.35–8.0 Hz]] muscles. The effect sizes of face yoga on improving the tonus were observed to have a moderate to significant effect in general. The highest effect size was reached significantly when the tonus of the digastric muscle was increased.

**Figure 3:**
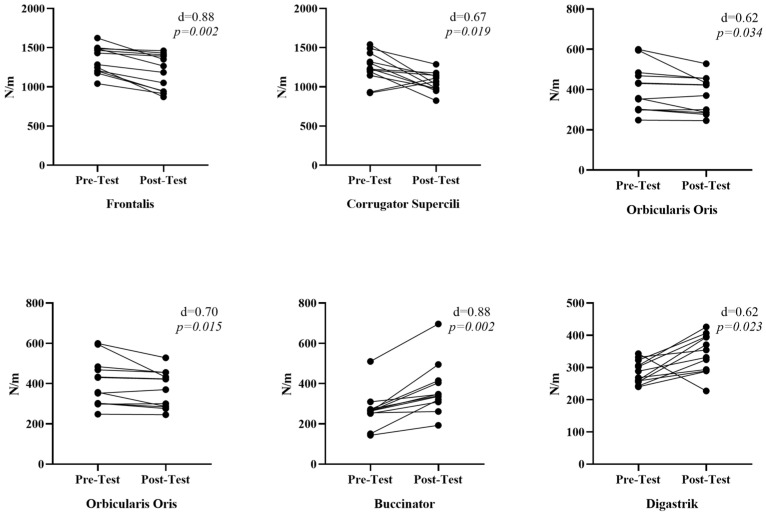
Comparison of facial muscles stiffness values pre- and post-face yoga.

According to the stiffness values (N/m) presented in Figure 3, a significant decrease in stiffness values was observed in the frontalis [r = 0.88, *p* = 0.002, 95% CI [−376.0–−26.0 N/m]], corrugator supercili [r = 0.67, *p* = 0.019, 95% CI [−590.5–180.5 N/m]], orbicularis oculi [r = 0.62, *p* = 0.034, 95% CI [−439.5–211.0 N/m]], and orbicularis oris [r = 0.70, *p* = 0.015, 95% CI [−163.0–18.0 N/m]] muscles. On the other hand, a significant increase was observed in the stiffness values of the buccinator [r = 0.88, *p* = 0.002, 95% CI [6.5–235.0 N/m]] and digastric [r = 0.75, *p* = 0.023, 95% CI [−15.75–133.5 N/m]] muscles. Regarding the effect size of face yoga in improving the stiffness of these muscles, we observed that it had a moderate-to-significant effect in general. In particular, the highest effect size was reached in increasing the stiffness of the digastric muscle.

**Figure 4:**
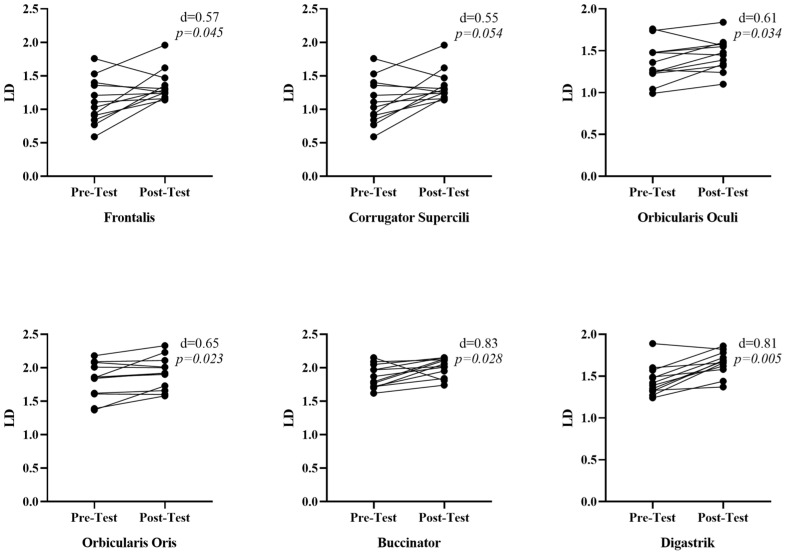
Comparison of facial muscle elasticity values pre- and post-face yoga.

According to the elasticity values (LD) presented in Figure 4, a significant increase in elasticity values was observed in all facial muscles. The frontalis [r = 0.57, *p* = 0.045, 95% CI [−0.29–0.69 LD]], corrugator supercili [r = 0.55, *p* = 0.054, 95% CI [−0.37–0.47 LD]], orbicularis oculi [r = 0.61, *p* = 0.034, 95% CI [−0.20–0.30 LD]], orbicularis oris [r = 0.65, *p* = 0.023, 95% CI [−0.07–0.37 LD]], buccinator [r = 0.83, *p* = 0.028, 95% CI [−0.34–0.36 LD]], and digastric [r = 0.81, *p* = 0.005, 95% CI [−0.07–0.39 LD]] muscles showed increased elasticity values. The effect sizes of face yoga in improving the elasticity of these muscles were observed, indicating that it had a moderate-to-significant effect in general. In particular, the highest effect size was reached in increasing the elasticity of the digastric muscle.

## 4. Discussion

This study investigated the effects of 8 weeks of intensive face yoga on the biomechanical properties of facial muscles in middle-aged women. The findings revealed that face yoga showed different effects depending on the physiological structure and function of the muscles. Superficial facial muscles showed decreased tonus and stiffness in response to relaxation-orientated exercises, which is considered an important finding in reducing excessive muscle tension. In contrast, the buccinator and digastric muscles, which are functional and dynamic, showed an increase in tonus and stiffness in response to isotonic contractions, indicating that face yoga may have a strengthening effect. In addition, the increase in elasticity observed in all muscles suggests that face yoga positively affects connective tissue and may reduce the loss of elasticity in aging.

Face yoga has recently become increasingly popular among non-surgical facial rejuvenation methods [9]. This method, which aims to strengthen facial muscles, increase skin elasticity, and reduce the effects of tense facial expressions, consists of exercises focusing on relaxing the face and balancing the tonus [26]. Facial exercises for the cheek muscles, mouth area, eyes, and under the chin are the most recommended to maintain the youthful appearance of the face [27]. In our study, we aimed to train both the superficial facial muscles that form facial expressions and the dynamic and functional muscles together by targeting the frontalis, corrugator supercilii, orbicularis oculi, orbicularis oris, buccinator, and digastric muscles, which are highly correlated with signs of aging, with the face yoga protocol that we created in line with these recommendations. Thus, we evaluated face yoga’s esthetic and potential other effects on muscle activity and functionality.

Although face yoga and similar facial exercises are thought to have the potential to improve skin appearance and reduce signs of aging, most of the studies in this field are based on subjective evaluations. Therefore, to definitively determine the effectiveness of face yoga, more comprehensive research is required to obtain more reliable and objective results [28,29]. Thus, in this study, the changes in tonus, stiffness, and elasticity in the facial muscles pre- and post-face yoga were measured with the Myoton^®^PRO device, which is increasingly accepted in the literature and allows for the objective assessment of muscle biomechanics [30].

During the aging process, the superficial facial muscles gradually flatten and shorten. This is related to the fact that repeated muscle contractions over the years both expel deep adipose tissue from below the muscle plane and increase the muscle resting tonus. As a result, the facial appearance becomes harsher and exhibits more pronounced signs of aging over time [31]. At this point, exercise can increase the maintenance of skin functions and rejuvenate its appearance by promoting mitochondrial biosynthesis [32]. Our 8-week intensive face yoga study found decreased tonus and stiffness in superficial facial muscles. The decrease in tonus and stiffness observed in the frontalis and corrugator supercili muscles shows that face yoga can reduce excessive tension caused by stress and repetitive mimic movements. It reveals the potential of face yoga in reducing wrinkles in the forehead and eyebrow area. The reduction in tonus and stiffness observed in the orbicularis oculi and orbicularis oris muscles shows that face yoga provides a more natural facial expression by reducing excessive tension in these areas. In addition, the increased elasticity in these muscles suggests that face yoga positively affects the subcutaneous connective tissue.

The buccinator muscle is located in the cheek region and participates in essential functions such as chewing, swallowing, and speaking. Unlike other facial muscles, the buccinator has a more functional and dynamic structure and is more exposed to isotonic contractions [33]. A study conducted on 16 participants aged 40–65 showed a significant increase (*p* = 0.002) in upper and lower cheek fullness and an average 2.7-year rejuvenation in the participants’ age perception after 20 weeks of facial exercises [12]. In our study, we showed that face yoga increased the tonus and stiffness of this muscle but also improved its elasticity. The main reason may be that face yoga’s pulling and resistance movements cause the muscle to contract actively, resulting in a hypertrophy effect. The increase in the tonus of the buccinator muscle suggests that, unlike other muscles, it responds with an increase in strength instead of relaxation. In our study, we started from the general relationships between biomechanical properties and power generation to correlate the increase in tonus and stiffness measured by Myoton^®^PRO with the increase in muscle strength. In the literature, it has been shown that tonus and stiffness increases occur in parallel with strength increases [34]. Therefore, interpreting an increase in tonus and stiffness as an increase in strength seems reasonable in light of the available information. In this context, the use of isotonic and isometric movements, especially for the lip and cheek area during face yoga exercises, may have caused an increase in the strength and tonus of this muscle. This suggests that face yoga is not only focused on relaxation but also has a muscle-strengthening effect [26].

In addition to the facial muscles that form facial expressions, our study evaluated the digastric muscle. Examining this muscle provided a more comprehensive investigation of the effects of face yoga on the lower face area. Aging leads to volume reduction and changes in fiber-type composition in the digastric muscle. These changes may affect the functional capacity and structure of the muscle [35,36]. Increasing the strength of the digastric muscle allows the skin to become firmer and more elastic, and it has been shown that increased muscle strength is directly related to improvement in skin elasticity [37]. Our study observed that the digastric muscle showed the most significant improvement after face yoga exercises, with an increase in the tonus and stiffness of this muscle and a substantial improvement in its elasticity. Movements based on stretching and isometric contractions for the chin area may cause both strengthening and increased muscle elasticity. The elasticity of the muscles in the neck and lower face area may also make the jawline more prominent over time [38].

At older ages, the elasticity of facial muscles decreases, leading to a loss of tissue elasticity and a marked decrease in the dynamics of mimic movements [37,39,40]. In a study investigating whether facial resistance exercises affect the mechanical properties of facial skin and skin aging, the skin became firmer and more elastic with exercise, and the elasticity of the orbicularis oculi and buccinator muscles increased significantly [37]. In our study, increased elasticity was observed in all muscle groups. This may be explained by fascia, connective tissue, and muscle cell adaptations. Exercise may allow muscle fibers to work more flexibly by increasing the mobility of superficial and deep fascia [41]. Mechanical loading may promote connective tissue remodeling by increasing fibroblast activity [42]. Specifically, repetitive mechanical stimuli activate cellular pathways that regulate fibroblast collagen synthesis and matrix remodeling [43]. Flexibility exercises may increase the range of motion by allowing muscle fibers to work in a more extended position [44]. These results suggest that face yoga may be a muscular exercise and a holistic method that improves connective tissue and skin elasticity.

In our study, superficial facial muscles and the buccinator and digastric muscles showed different responses in terms of tonus and stiffness. This other response may be because the buccinator and digastric muscles act as postural and functional muscles. As functional muscles require continuous use, exercises targeting them usually increase the tonus and strength [45]. In contrast, since superficial facial muscles work more for facial expressions, they may respond better to relaxation-orientated movements [46]. In addition, the anterior belly of the buccinator muscle and the digastric muscle contain more type II (fast twitch) fibers and work actively in movements requiring force [47,48]. Therefore, an increase in tonus and stiffness can be observed during face yoga. Other facial muscles contain more type I (slow twitch) fibers associated with facial expressions [48,49]. During face yoga, these muscles lose their excessive tension with relaxing and stretching movements, which may decrease the tonus and stiffness. These physiological differences explain the different effects of face yoga on different facial muscles. Therefore, face yoga should be designed individually, and exercises for other muscle groups should be carefully selected.

It is known that BMI, which tends to increase with age in women, may affect facial soft tissue characteristics [50]. However, the direct effect of BMI on facial muscle activity or response to exercise has not yet been demonstrated in the existing literature. Cotofana et al. [51] reported that BMI had no significant effect in their study examining facial muscle activity in aging. In our study, since all participants were in a similar BMI range and individual changes were evaluated in pre-post measurements, the effect of BMI as a confounding factor was considered limited. However, it is recommended that BMI stratification or skin thickness measurements be included in future studies to obtain more precise results.

One of the most important strengths of this pre-experimental clinical trial is that muscle tonus, stiffness, and elasticity were measured objectively and reliably using the Myoton^®^PRO device. This increased the reliability and validity of the findings obtained in the study. In addition, the fact that the practice covered 8 weeks and the participants regularly exercised face-to-face supports the consistency of the findings. In addition, we have the following limitations. Firstly, the findings should be considered preliminary data only due to this study’s pre-experimental design. The study was conducted in a small sample group (n = 12), which limits the generalizability of the findings. Secondly, no control group was used in the study. Therefore, factors other than face yoga may influence the results obtained. Over the eight weeks, factors such as natural biological variations, seasonal effects, the Hawthorne effect (behavioral effects in participants knowing that they were being observed), changes in hydration/circulation, or variations in measurement technique may have contributed to the results. Thirdly, the fact that the practice covers 8 weeks prevents the evaluation of long-term effects. The fact that the participants were only female academic staff, a particular group that may differ from the general population regarding lifestyle, stress levels, health awareness, and adherence, and between the ages of 45 and 55, limits the generalizability of the findings to different ages and genders and other populations. Finally, our study focused on statistical significance and effect sizes, but there was no further discussion of the observed changes’ clinical significance or practical relevance. This is an important point, and we think that future studies should include subjective satisfaction interrogations and questionnaires to assess whether esthetic improvements or functional benefits perceived by individuals are clinically meaningful. Furthermore, monitoring home program compliance by participant self-reporting is a limitation of our study, which may affect the interpretation of the results. In future studies, the reliability of the conclusions can be increased by using a larger sample group and control group. In addition, longer-term studies are recommended to evaluate the long-term effects. Studies involving different age and gender groups will allow the impact of face yoga to be assessed in the broader population. Furthermore, potential objective monitoring methods (e.g., digital monitoring, logbooks with specific details, randomized supervised controls) are suggested for future studies. In addition, our study used general reliability information based on the available literature on the Myoton^®^PRO device’s accuracy and reliability for facial muscles. However, we think that more confirmatory evidence for these specific muscles would be helpful, and we recommend collecting more data on this in future studies. Electromyographic (EMG) analyses are also recommended for a more comprehensive evaluation of the effects of face yoga on muscle activation, and histological and biochemical studies are recommended for a more detailed examination of changes in the physiological structure of muscles.

## 5. Conclusions

This pre-experimental clinical trial shows that face yoga can change the biomechanical properties of specific facial muscles in middle-aged women. While relaxation and increased elasticity were observed in superficial facial muscles, tonus and elasticity increased in functional muscles. These findings suggest that face yoga may have selective effects depending on muscle physiology, provide relaxation, and create a strengthening impact on specific muscle groups. In addition, it is predicted that face yoga can support facial muscles’ function and esthetic appearance in the aging process. However, more comprehensive randomized controlled studies are needed to evaluate the generalizability of these findings.

## Figures and Tables

**Table 1:** The face yoga program.

Diaphragmatic Breathing	6 s Break, 3 Repetitions
Neck Stretching Exercises Stretching the side neck muscles: While one hand grasps the side of the chair, the other hand grasps the side of the head from the side, and the other side stretches the side neck muscles. Stretching the back diagonal neck muscles: While one hand grasps the side of the chair, the other hand grasps the front of the head. The back diagonal neck muscles are stretched by pulling the head slightly forward and to the other side. Stretching the back neck muscles: Both hands grasp the top of the head and tilt the head forward, stretching the back neck muscles.	Right–left side, 6 s break, 2 repetitions. Count to 10 in all exercises.
Neck Isometric Exercise On the right side, the right hand is placed on the right side of the head; on the left side, the left hand is placed on the left side of the head. The head and hand are pushed towards each other for 10 s. Then, both hands are placed on top of each other, both on the front and back of the head. The head and hands are pushed towards each other for 10 s.	6 s break, 2 repetitions
Shoulder Roll Exercise	6 s break, 2 repetitions
Neck Massage	Massage 30 times (6 s break, 2 repetitions).
Double-Chin Exercise	6 s break, 3 repetitions
Lip Exercise	Open and close the mouth 10 times 6 s break, 3 repetitions
Cheek and Lip Exercise “a” sound, ‘o’ sound, ‘i’ sound, and ‘u’ sound are made	6 s break, 2 repetitions
Cheek and Lip Exercise	Cheeks and lips are filled with air, 6 s break, 3 repetitions
Cheek and Lip Exercise	Lips are pushed forward as if making a kiss, 6 s break, 3 repetitions
Face Massage	3 repetitions
Lip Exercise	6 s break, 3 repetitions
Lip and Nose Massage	6 s break, 6 repetitions
Eye Contour and Crow’s Foot Exercise	6 s break, 3 repetitions
Under-Eye Exercise	6 s break, 3 repetitions
Eyelid Exercise	6 s break, 3 repetitions
Eye Contour Exercise	6 s break, 3 repetitions
Eyebrow Center Exercise	6 s break, 3 repetitions
Eyebrow Center Massage	6 s break, 3 repetitions
Forehead Exercise	6 s break, 3 repetitions
Forehead Massage	2 repetitions
Full Face Exercise	6 s break, 3 repetitions

## Data Availability

The datasets generated and/or analyzed during the current research are available from the corresponding author on reasonable request.

## Supplementary Materials

The following supporting information can be downloaded at: https://www.mdpi.com/article/10.3390/medicina61050840/s1.

## Abbreviations

The following abbreviations are used in this manuscript:

TMJ	temporomandibular joint
BMI	Body Mass Index
Sec	second
