# Supplementary material for: Effect of Intensive Face Yoga on Facial Muscles Tonus, Stiffness, and Elasticity in Middle-Aged Women: A Pre-Experimental Clinical Trial
Source: Medicina (Kaunas). 2025 May 2;61(5):840. doi: 10.3390/medicina61050840 (PMC12112979; doi:10.3390/medicina61050840)
Supplement: Supplementary file 1 [file medicina-61-00840-s001.zip › medicina-3611545-supplementary.pdf]

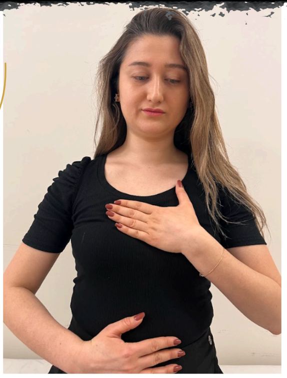

Diaphragmatic Breathing

Inhale deeply through the nose, expand your belly, count to 6-10, and hold and exhale through the nose again.

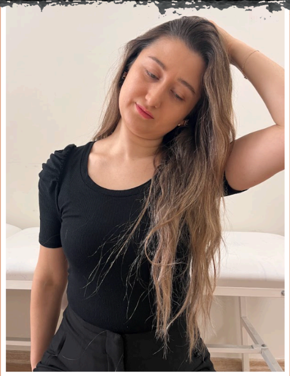

Stretching the Side Neck Muscles

With one hand grasping the side of the chair and the other hand grasping the side of your head, gently pull your head to the other side and stretch the side neck muscles. Count for 10 seconds.

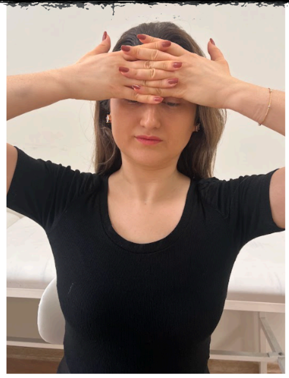

Neck Isometric Exercise

Place both hands on each other and place them on the forehead. Push your head and hands towards each other for 10 seconds.

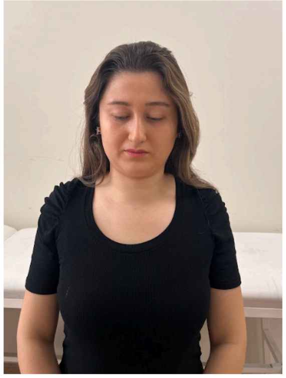

Shoulder Roll Exercise

Roll your shoulder from front to back and back to front 10 repetitions.

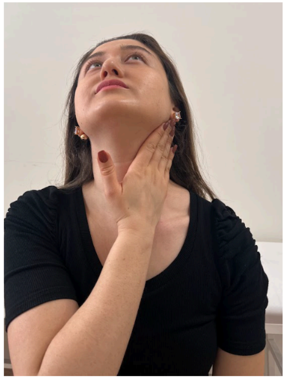

Neck Massage

Before starting, apply oil to your neck. Tilt your head back and massage your neck from bottom to top—never from top to bottom. Repeat 30 times.

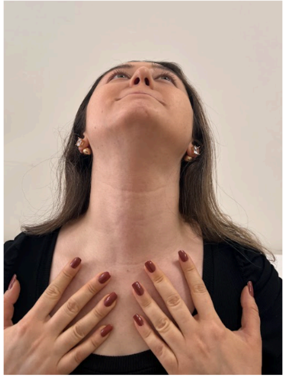

Double Chin Exercise

Take a deep breath through your nose, tilt your head back, place your fingers on your cleavage area, and pull down on this area while pulling your lower lip towards the tip of your nose, and stay in this pose for 10 seconds.

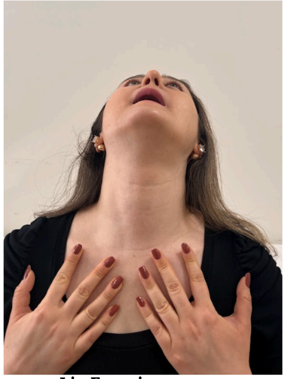

Lip Exercise

Place your fingers on your cleavage and pull down. Curl your lips towards your mouth, take a deep breath through your nose, tilt your head back, and open your mouth as slowly as possible. Continuing the movement, exhale through your mouth and slowly close your mouth. Slowly open your mouth again without closing it completely. Open and close your mouth 10 times.

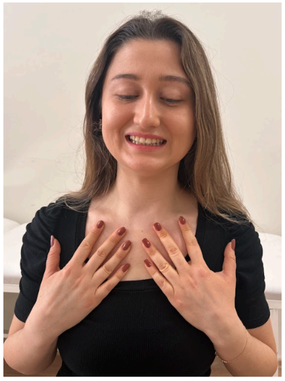

Cheek and Lip Exercise

Take a deep breath through your nose, open your mouth as if making the sound iii.... and hold this pose for 3 seconds.

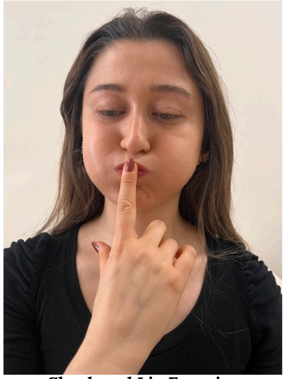

Cheek and Lip Exercise

Inflate by filling your mouth (around the cheeks and lips) with air and waiting 20 seconds. At the same time, keep your index finger pressed lightly on your lips, then slowly release the air.

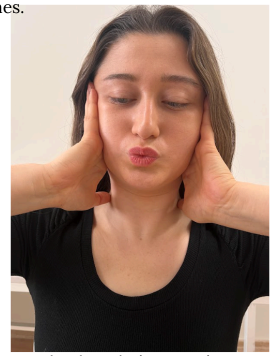

Cheek and Lip Exercise

Take a deep breath, place your hands on your cheeks (near the end of your ears), and pull them back; at the same time, push your lips forward like a kiss and slowly release the breath from your mouth. Stay in this pose for 10 seconds.

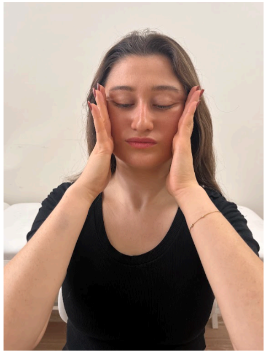

Face Massage

Place your hands on the jawbone joint under the ear and massage the cheeks 3 times with circular movements from outside to inside. Bring your hands together over your nose, pull both hands together downwards, bring your fingertips under your chin, then stroke from under your chin towards your ears.

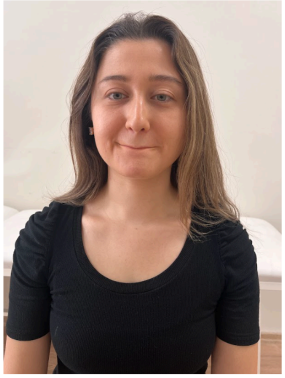

Lip Exercise

Take a deep breath, pull your lips inward, and hold the pose for 3 seconds. Then exhale and forcefully say PE, pushing your lips outward.

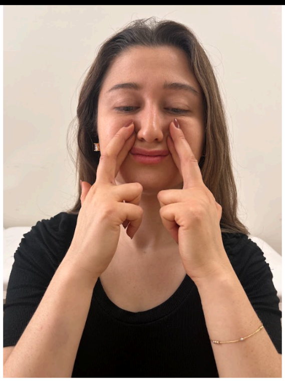

Lip and Nose Massage

Place the middle fingers on the index fingers, open your mouth slightly, start from the corner of the mouth, and massage the entire line upwards. Finish the massage on the nose and repeat 6 times.

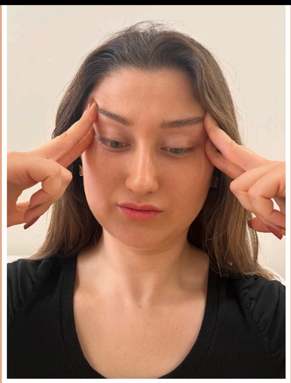

Eye Contour and Crow's Feet Exercise

Place the index fingers at the end of the eyes and the middle fingers at the end of the eyebrows and pull them back. At the same time, squint your eyes and stay in this pose for 10 seconds.

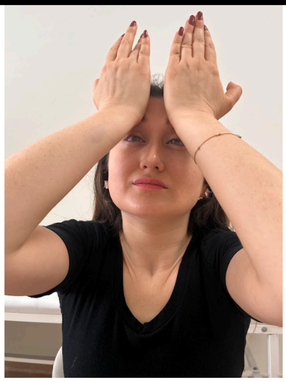

Under Eye Exercise

Place the starting point of your wrists where your eyebrows start and pull your eyebrows up while focusing on your lower eyelids and trying to pull them up. Stay in this pose for 10 seconds.

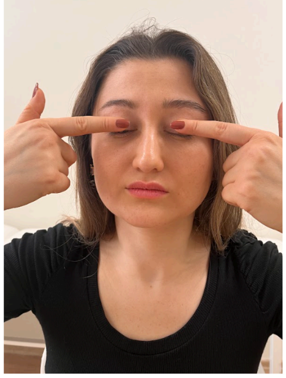

Eyelid Exercise

Place your index fingers on your eyelids and try to open your eyes (without pressing). Stay in this pose for 10 seconds.

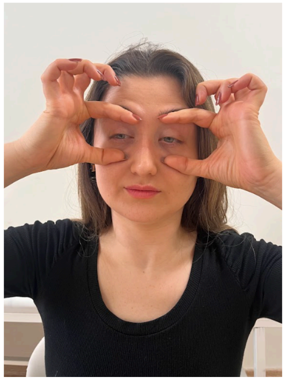

Eye Contour Exercise

Place your index fingers under your eyebrows and your thumbs under your eyes. Open your eyes with your fingers, squint your eyes simultaneously, and wait 10 seconds.

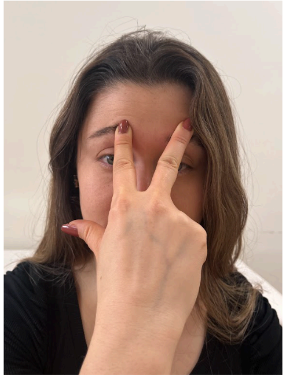

Eyebrow Center Exercise

Use your middle and index fingers to pull your eyebrows apart, try to frown simultaneously, and hold this pose for 10 seconds.

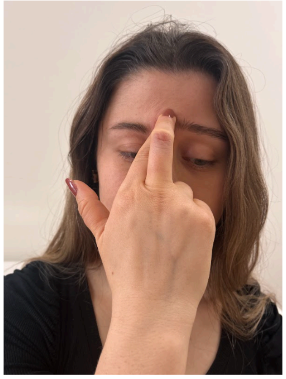

Eyebrow Center Massage

Apply oil to the center of the eyebrows. Place your middle finger on your index finger, and massage the index finger between the eyebrows in a circular motion 10 times clockwise and 10 times counterclockwise.

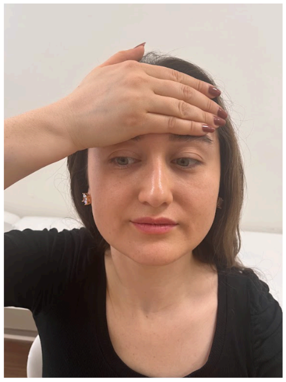

Forehead Exercise

Place one hand on your forehead (do not press), and raise your eyebrows. Your hand will remain still, just preventing the eyebrows from raising. Hold this pose for 10 seconds.

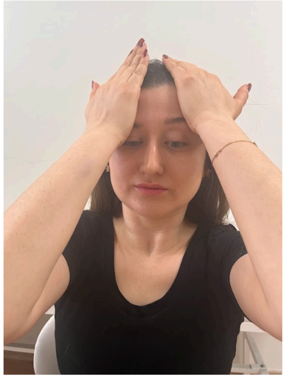

Forehead Massage

Place your hands on your forehead. While one hand is still, massage with the other hand in a clockwise direction 10 times and in the counterclockwise direction 10 times.

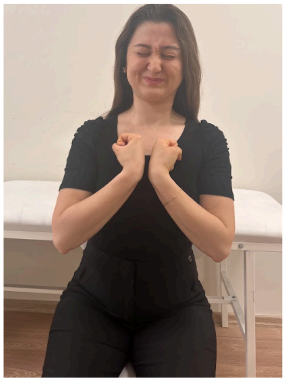

Full Face Exercise-A

Breathe slowly through the nose, make fists with your hands, and clench them. Close your eyes and mouth and clench them for 10 seconds.

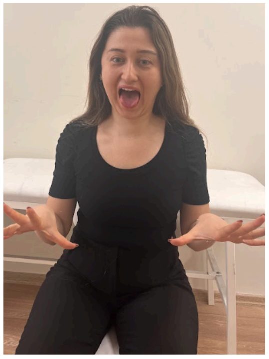

Full Face Exercise-B

Open your mouth as wide as you can as if you were saying the letter “a”, stick out your tongue, and extend it downwards. Open your eyes very wide and look at the farthest point. Open your fingers as wide as you can and exhale through your mouth. Stay in this pose for 10 seconds.
